# Aquaporins: Potential Targets in Inflammatory Diseases

**DOI:** 10.5152/eurasianjmed.2023.23357

**Published:** 2023-12-01

**Authors:** Ayse Bozkurt, Hamza Halici, Muhammed Yayla

**Affiliations:** 1 Department of Pharmacology, Van Yüzüncü Yıl University Faculty of Pharmacy, Van, Turkey; 2 Department of Pharmacology, Atatürk University Hınıs Vocational College, Erzurum, Turkey; 3 Department of Pharmacology, Kafkas University Faculty of Medicine, Kars, Turkey

**Keywords:** Aquaporin, disease, inflammation, membrane, water

## Abstract

Inflammation involves a long chain of molecular reactions and cellular activity designed to repair tissue damaged by various causes. The inflammatory process and its complex mechanisms have recently become a focus of interest for many researchers. After the onset of inflammation, various adverse conditions that initiate the inflammatory response need to be addressed; however, failure to limit the inflammatory reaction may result in the damage or destruction of host cells. Therefore, inflammatory reactions play a role in many different diseases. Aquaporins (AQPs), commonly referred to as water channels, are protein channels responsible for forming pores in the membranes of biological cells. Their main function is to aid in the movement of water between cells. Aquaporins not only regulate transepithelial fluid transport across membranes but also play a role in regulating essential events crucial for the inflammatory response. Aquaporins have been shown in many studies to have important roles in inflammatory diseases. This clearly indicates that AQPs may be potential targets for inflammatory diseases. This review summarizes the research to date on the structure and function of AQPs and provides an update on the relationship between AQPs and various human inflammatory diseases.

## Introduction

The inflammatory process and the complex mechanisms involved in its regulation have been the focus of scientific interest for many researchers in recent years. Inflammation involves a long chain of molecular reactions and cellular activities designed to repair tissue damaged by various causes. The inflammatory process involves a series of events, including dilation of venules and arterioles, increased blood vessel permeability, and the migration of leukocytes into tissues. The immune system plays a pivotal role in recruiting plasma proteins and leukocytes to the relevant tissue site. However, when the inflammatory process becomes prolonged and persistent, it can result in maladaptive responses.^1^ Failure to resolve this can lead to tissue destruction, chronic inflammation, and progressive fibrosis.^2^ Persistent and excessive inflammation causes disruption of cell homeostasis.^3^ One of the clearest signs of impaired homeostasis is an increase in extracellular fluids (edema).^4^ This may cause some dysfunction in tissues and organs.^5^

Aquaporins, often known as water channels, are protein channels that create pores in the membranes of biological cells. Their main function is to aid in the movement of water between cells.^6^ Besides water, AQPs also enable the transport of glycerol, urea, and, in certain instances, ions.^7^ Mammals express 13 AQPs, each with different cellular and tissue expression and specific subcellular localizations.^8^ The 13 mammalian AQPs are broadly divided into 3 groups. AQP0, AQP1, AQP2, AQP4, AQP5, AQP6, and AQP8 are classified as classical AQPs involved in water transport. AQP3, AQP7, AQP9, and AQP10 are aquaglyceroporins that carry both glycerol and water. AQP11 and AQP12 differ from other AQPs in their localization. These AQPs are localized in the intracellular endoplasmic reticulum (ER). Here, osmotic changes and oxidative stress may contribute to ER homeostasis upon.^9^ The physiological role of different AQPs is affected during inflammation by mediators that regulate inflammatory diseases by inducing a number of changes in aquaporins.^10^

Many studies have shown that AQPs have important roles in inflammatory diseases.^10,11^ This clearly shows that AQPs may be potential targets for inflammatory diseases. In this review, the relationship between various inflammatory and AQP diseases is summarized.

### Aquaporins

Aquaporins, commonly known as “water channels,” are compact integral membrane proteins that aid in the facilitation of water transport across the membranes of diverse organ systems, including the kidney, central nervous system (CNS), lung, heart, eye, and skin.^12^ The transport of water between tissues and cells is a fundamental process that contributes to the maintenance of physiological homeostasis. Typical transcellular water transport can occur through passive diffusion with other solutes or by traversing the lipid layer of the membrane. Aquaporin water channels are located in regions where high water permeability is required to transport liquids. To accommodate these changing fluid transport processes, AQPs exhibit different cell and tissue distributions in different organ systems. The discovery of AQPs by Peter Agre of Johns Hopkins University in 1992 earned him the Nobel Prize in Chemistry in 2003.^13^ Aquaporins are commonly considered to play a role in diseases characterized by alterations in water transport.^14^

### Types and Functions of Aquaporins

Aquaporins are transmembrane water transport proteins found in many human tissues.^15^ The plasma membrane forms the main barrier to water transport. AQPs are important proteins that regulate water permeability across the plasma membrane. Thirteen members of the AQP family, ranging from AQP0 to AQP12, have been found in various mammalian cells^16^ (Table 1). The AQP family includes 3 subgroups: AQP, aquaglyceroporin, and superaquaporin. AQP0, AQP1, AQP2, AQP4, AQP5, AQP6, and AQP8 are only responsible for water transport, while aquaglyceroporin, which includes AQP3, AQP7, AQP9, and AQP10, absorbs only water and small water-soluble substances such as urea and glycerol. Aquaglyceroporins include the superaquaporins AQP11 and AQP12.^17^ While many of the AQPs are found at the plasma membrane to contribute to water transport due to osmotic gradients, the superaquaporins AQP11 and AQP12 are expressed in the cytoplasm, playing a role in regulating organelle volume, intracellular water transport, or vesicular homeostasis.^18^ Most human AQPs are distributed in different normal tissues, including endothelium and epithelium, and also in various other cell types, including astrocytes, erythrocytes, skeletal muscle, and adipocytes.^15^ AQP0 is present in the human lens and is involved in lens transparency and homeostasis.^19^ AQP1 is found in red blood cells, lungs, brain, and kidneys, playing a role in both water reabsorption and fluid secretion. AQP2 is predominantly found in the kidney, and mutations in the protein are associated with diabetes insipidus.^20^ AQP3 is found in various organs, such as the conjunctival epithelium, renal collecting duct, skin, and eyes.^21^ AQP4 is the predominant water channel expressed in the spinal cord, brain, and optic nerve, regulating the homeostasis of the brain.^22^ AQP5 is primarily found in the upper bronchus, alveolar epithelium, and trachea.^23^ Studies on AQP6 have unveiled that this protein is found in the carries, mainly nitrate, the membranes of intracellular vesicles, and is permeable to some ions, such as chloride ions, in the renal tract.^23^ AQP7 molecules are mainly expressed in skeletal muscle, heart, adipose tissue, testis, and kidneys and play a role in solute as well as water permeability.^24^ AQP8 is expressed in various tissues and organs, especially the lung, liver, stomach, kidneys, pancreas, salivary glands, testicles, epididymis, duodenum, jejunum, placenta, and trachea.^25^ AQP9 has been found in the brain, liver, ovary, leukocytes, and testis.^23^ AQP10 is selectively localized to the jejunum and duodenum but not to other parts of the gastrointestinal tract.^26^ AQP11 is found in the heart, brain, kidneys, liver, intestine, testes, and adipose tissue.^27^ but its function remains unknown. Although the specific location of AQP12 has not been determined, it is found only in pancreatic acinar cells.^28^

### Molecular Structure of Aquaporins

Aquaporin monomers weigh approximately 28-30 kDa each, and each one is composed of 6 capped α-helical domains, creating a barrel-shaped structure that spans the plasma membrane. The aquaporin polypeptide structure comprises a single chain of approximately 270 amino acids, with amino (N) and carboxyl (C) terminals located in the cytoplasm.^29^ A shared characteristic among all AQPs is the presence of the asparagine–proline–alanine (NPA) consensus motif, which is widely believed to play an important role in pore formation.^30^ Two sets of highly conserved motifs, each containing a short helix with the sequence NPA motifs, are positioned on opposite sides of the monomer. The AQP monomer possesses independent water pores and forms homotetramers to facilitate the transport of liquids. By combining AQP monomers, homotetramers form the central pore. Many studies have demonstrated that the central pore in AQP1, AQP4, and AQP5 is permeable to nitric oxide, CO_2_, or O_2_ gases. Gas transport by AQPs is faster than free diffusion, so their role in biological functions is important.^31^

### Inflammatory Diseases and Aquaporins

**Figure 1.**
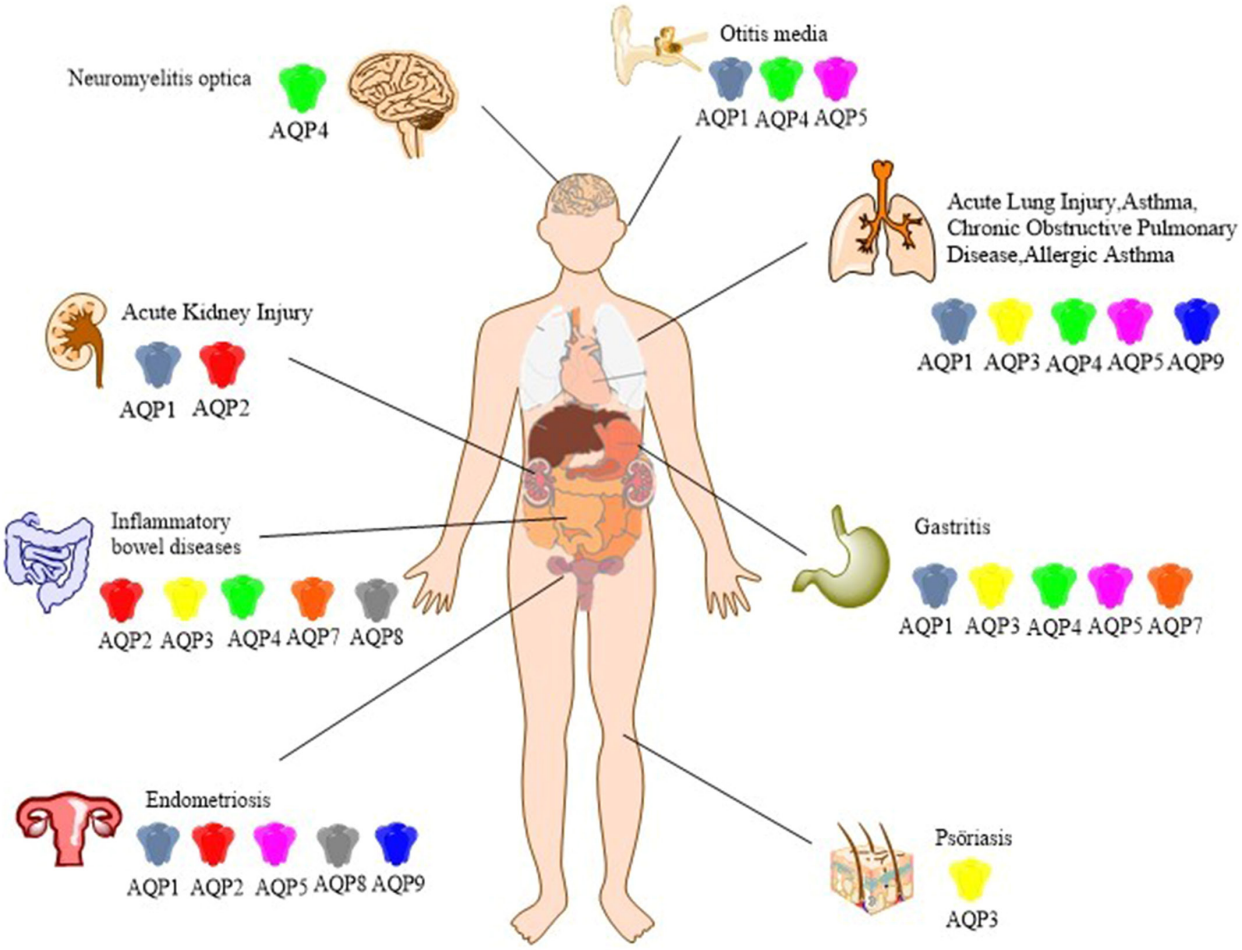
Location of different aquaporin subtypes in the body.

Inflammation is a natural reaction to detrimental stimuli, including damaged cells or pathogens, toxic compounds, and irradiation, thus acting by initiating the healing process and eliminating harmful stimuli.^32^ Throughout the inflammatory process, macrophages induced by inflammation play a crucial role in supervising various immunological events. This includes the overproduction of inflammatory mediators, an excessive production of oxidants, and pro-inflammatory cytokines.^33^ Lately, many inflammatory biomarkers have been discovered, including acute phase protein, platelet-activating factor, prostaglandins, kinins, purines, amines, leukotrienes, cytokines, adhesion, chemokines, and nuclear factor kappa B (NF-κB), Mitogen-activated protein kinase (MAPK), and JAK-STAT pathways.^2^ Also, it is considered to play a crucial role in the pathogenesis of inflammation by inducing NF-κB activation and the expression of many pro-inflammatory molecules.^34^ Once this process begins, the goal is to eliminate the pathogen. However, if the inflammatory response cannot be sufficiently controlled, it could result in the detriment of host cells.^35^ Therefore, inflammatory reactions play a role in many different diseases, and the pharmacological control of inflammation in various organs will continue to be a focus in the development of new drugs. Therefore, we examined the relationship between AQPs and inflammatory diseases in the various organs shown below (Figure 1).

### Aquaporins in Acute Lung Injury

Acute lung injury (ALI) is a lung disorder characterized by damage to the alveolar capillary walls, edema and infiltration of inflammatory cells.^36^ Lung injury is pathologically defined by widespread alveolar damage, ranging from ALI to the more severe condition known as acute respiratory distress syndrome (ARDS).^37^ Traditionally, certain treatment approaches have been employed as supportive strategies in ARDS and ALI, but their efficacy has been limited, resulting in a persistently high mortality rate. As of now, no definitive measures to tackle the challenge of long-term mortality have been identified.^38^ To investigate further therapeutic approaches, current research is dedicated to preventing or alleviating the advancement of lung injury. This involves utilizing strategies grounded in the contemporary comprehension of biological mechanisms at the molecular level. It is hypothesized that ALI can be prevented or alleviated by employing pharmacological agents that either impede the generation of reactive oxygen species (ROS) or neutralize these reactive molecules.^39^ Experimental studies such as liposaccharide (LPS), hydrochloric acid, hyperoxia, and ventilation have been used to examine the mechanisms of lung damage and the contribution of AQP in this process. Pretreatment of Nervilia fordii (NF) can promote the upregulation of pulmonary AQP-1 and AQP-5 expression, increase clearance and water transport in the lungs to improve fluid balance, and reduce pulmonary edema to effectively preserve the lungs from acute injury.^40^ It was shown that a calcitonin gene-related peptide (CGRP) 8-37 antagonist could ameliorate LPS-induced ALI in a mouse model by modulating the expression levels of AQP-1 and AQP-5 by reducing inflammatory cytokines.^41^ Furthermore, a later study using a mouse model of oleic acid-induced ALI showed that AQP4 mRNA was overexpressed in type II alveolar epithelial cells.^42^ Tanshinol, an aqueous polyphenol, was shown to increase AQP5 expression by inhibiting tumor necrosis factor (TNF)-α and interleukin 6 (IL-6) and reducing p38 phosphorylation in mouse lung tissue in a cecal ligation and puncture (CLP)-induced sepsis model.^43^

### Aquaporins in Sepsis

Sepsis, commonly known as systemic inflammatory response syndrome, arises from bacterial infections or other factors that trigger immune responses in the host.^44^ In sepsis, there is an overproduction of oxygen-free radicals, leading to an imbalance in the natural scavenging mechanisms. These processes are implicated in microvascular dysfunction, followed by organ dysfunction.^45^ The cardiovascular and respiratory systems are predominantly affected by acute organ dysfunctions in sepsis, with a particular emphasis on the lung as a critical organ.^46^ Sepsis is of significant concern; multiple organ dysfunction syndrome develops in 30% of these cases and occurs in 10 out of 1000 hospitalized patients. Mortality is noted in 20% of individuals diagnosed with sepsis, and this rate escalates to 60-80% among those who progress to septic shock. Timely diagnosis and intervention are crucial, given the elevated mortality rate.^47^ Despite the high morbidity and mortality associated with sepsis, delays in diagnosing this condition and its related complications persist.^48^ Considering the intricate nature of sepsis pathogenesis, it is widely recognized that immune cells, specifically monocytes, macrophages, and neutrophils, play a central role.^49^ The substantial gathering of neutrophils in lung tissue can impact inflammatory processes, leading to an increased production of various inflammatory cytokines, including ILs and TNF.^50^ These factors are believed to play a crucial role in the pathogenesis of sepsis.^51^ The NF-κB is an inducible nuclear transcription factor that centrally regulates the transcription of various genes, including those encoding adhesion molecules, proinflammatory cytokines, and other proinflammatory mediators implicated in septic shock and severe sepsis.^52^ Furthermore, evidence suggests that oxidative stress may play an important role in the organ damage and mortality caused by sepsis.^53^ One study reported that in CLP-induced sepsis, elevated oxidative stress in tissues, along with plasma, is a crucial mechanism attributed to the release of free radicals.^54^ To enhance comprehension of the mechanisms underlying clinical sepsis and develop more effective treatments, various models, including CLP, are utilized for the induction of polymicrobial sepsis. The CLP is a commonly used model for polymicrobial septic shock and is considered one of the most favored animal models for sepsis.^55^ Moreover, models involving high-dose LPS administration as well as mesenteric ischemia and reperfusion are employed.^56^ While numerous new mechanisms are under investigation to enhance our understanding of sepsis pathophysiology, currently, there is no recognized and effective treatment protocol. In the literature, a substantial body of experimental and clinical research is dedicated to exploring the treatment and pathophysiology of sepsis, underscoring the significance and popularity of this issue.^57^ Hence, to completely comprehend the role of AQPs in sepsis, it is crucial to understand how their expression is modified during inflammation. One study showed that AQP1 expression increased twofold and AQP3 expression decreased 2.5-fold in the leukocytes of septic patients.^58^ In a CLP mouse model of sepsis, it was demonstrated that AQP2 expression is reduced through the NF-κB pathway, potentially contributing to acute renal failure during sepsis.^59^ In a study conducted on septic encephalopathy (SE), it was shown that brain inflammation, mediated by neutrophil infiltration, leads to the upregulation of AQP4, exacerbating brain edema.^60^ Studies with tanshinol and emodin^61^ have shown that they can upregulate the expression of AQP-5 by inhibiting inflammatory cytokines, thus protecting the lung tissue of rats with sepsis.^43^ In rats subjected to sepsis through CLP, the assessment of liver AQP8 expression revealed a marked decline in the AQP8 protein level within the canalicular membranes. Notably, there was no significant reduction observed in the expression of AQP9.^62^

### Aquaporins in Allergic Rhinitis–Asthma–Chronic Obstructive Pulmonary Disease

Allergic rhinitis (AR) is a chronic disease that influences 40% of the global population and is characterized by various inflammatory mediators such as histamine. The frequency of AR continues to increase worldwide. Aquaporins are a family of small membrane proteins that preserve airway fluid homeostasis and interact with asthma, airflow obstruction, pulmonary edema, inflammation, and other environmental factors.^63^ *Juglans regia* was shown to improve allergic asthma by inhibiting inflammatory cytokines and elevating protein expression levels of AQP-4, and AQP-5.^64^ Another study stated that AQP3 potentiated ovalbumin-induced mouse asthma by facilitating H_2_O_2_ membrane permeability, increasing chemokine production (CCL22 and CCL24), and T cell trafficking from alveolar macrophages.^65^

For individuals diagnosed with chronic obstructive pulmonary disease (COPD), airway inflammation related to the infiltration of neutrophils, macrophages, and T lymphocytes and elevated levels of inflammatory cytokines are observed. Oxidative stress plays an significant role in this inflammatory state. Chronic inflammation may cause spasms, narrowing of the bronchial walls, and hyperplasia.^66^ In one study, with the deletion of AQP5, increased mucus secretion and higher concentrations of the protein were found in the upper respiratory tract of mice.^67^ Treatment with hydrogen-rich saline reversed the decrease in AQP5 levels in COPD rats, indicating its potential for regulating mucus hypersecretion through the modulation of AQP5 expression.^68^

### Aquaporins in Psoriasis

Psoriasis is a chronic inflammatory skin condition identified by dysregulated skin immune responses and impaired skin barrier function.^69^ Immune cells produce various inflammatory cytokines such as IL-22 and IL-17, which directly interact with the cells, causing psoriatic inflammation and thereby causing the immune response to cause local inflammation and keratinocyte proliferation. A study conducted by Hara-Chikuma et al^70^ on AQP3-deficient keratinocytes and AQP3-deficient mice revealed an additional role of AQP3 in the pathogenesis of psoriasis by correlating the trigger of the NF-κB pathway via extracellular HO uptake with AQP3 channel activity. When Lee et al^71^ compared healthy skin with the skin of psoriasis patients, they found that AQP3 protein expression was reduced in perilesional and damaged skin.

### Aquaporins in Otitis Media

Otitis media (OM) denotes inflammation of the middle ear (ME), irrespective of the underlying mechanism or cause.^72^ Alterations in the water and electrolyte composition within the mucosa of the ME may give rise to otorrhea and ME effusion, potentially contributing to the onset and persistence of ME diseases, including OM.^6^ Eleven AQP types, ranging from AQP1 to AQP11, have been identified to be expressed in the ME and eustachian tube through techniques such as immunohistochemistry or Western blotting. Earlier investigations of the human ME have detected AQP5 in the ME mucosa, AQP3 and AQP10 in otitis media with effusion, and AQP1, 2, 3, 4, 5, 6, 8, 10, and 11 in the normal human ME. Nonetheless, limited research has been conducted to investigate the function and role of AQPs in acute and chronic otitis media. Aquaporins are believed to play a role in water regulation, and alterations in the expression patterns of various AQPs, particularly AQP1, AQP4, and AQP5, have been noted in response to inflammatory stimuli such as LPS. This implies that AQPs might possess immunological functions in OM.^73^

### Aquaporins in Inflammatory Bowel Diseases

Enteritis is marked by aberrant water and electrolyte transport phenomena, coupled with inflammation and damage to the intestines.^74^ In the early stages of inflammatory bowel disease (IBD), a diminished mRNA expression of several AQPs, such as AQP1, AQP3, AQP7, and AQP8, has been evidenced in the human intestinal mucosa, encompassing ulcerative colitis and Crohn’s disease. Consequently, apical AQP8 immunolabeling is diminished in the surface epithelium or crypts in the colon of IBD patients.^75^ In the human colon, AQP3 expression is primarily observed in mucosal epithelial cells, as indicated by a particular study.^76^ Moreover, findings indicate that AQP7 and AQP8 are situated on the apical side, contrasting with AQP3’s basolateral localization. In the context of allergic diarrhea, Yamamoto et al^77^ identified a reduction in AQP4 and AQP8 expression in the colon. Therefore, colon AQPs are novel therapeutic targets for diarrhea and constipation. For example, Camilleri et al^78^ found reduced AQP3 mRNA and protein abundance in reconstituted rectal bacteria, predominantly with irritable bowel syndrome, regardless of the presence or absence of bile acid malabsorption, compared with a healthy control group. Hence, AQP3 is believed to have a crucial role in water transport within the colon. Nevertheless, the physiological role and regulation of AQP3 expression are not fully understood. The analysis of AQP3 in the colon is thought to hold promise for the development of novel methods and treatments aimed at preventing diarrhea and constipation.^79^

### Aquaporins in Neuromyelitis Optica

Neuromyelitis optica (NMO), mainly targeting the optic nerve and spinal cord, is characterized as an autoimmune disorder affecting the central nervous system.^80^ The discovery of autoantibodies against AQP4-IgG has transformed the understanding of the immunopathogenesis of NMO, revolutionizing the diagnostic process. Immunoglobulin G antibodies against AQP4-IgG were positive in the majority of cases. AQP4 is a water channel protein highly prevalent in the CNS and is found in the terminal processes of astrocytes. Autoreactive B cells from individuals with neuromyelitis optica spectrum disorder (NMOSD) can produce AQP4 IgG following IL-6 stimulation in conjunction with CD4+ T cells. Activated B and T lymphocytes have the capability to generate inflammatory cytokines, traverse the brain microvascular endothelium, and compromise the blood–brain barrier, ultimately resulting in the infiltration of AQP4-IgG and other immune cells like granulocytes and macrophages into the CNS.^81^ The identification of AQP4-IgG and the comprehension of NMOSD immunobiology have led to the evolution of clinical approaches involving monoclonal antibodies against cytokine receptors, B cells, and activated complement. However, additional research is imperative due to a lack of information regarding pathogens and immunogenicity in seronegative patients. In the future, new treatments based on other immune targets may be developed to obstruct NMOSD attacks with minimal side effects and lower the risk of permanent disability.^82^

### Aquaporins in Endometriosis

Endometriosis is a chronic, hormone-dependent, and inflammatory condition distinguished by histological lesions caused by the presence of endometrial tissue beyond the limits of the uterine cavity.^83^ Particularly, 9 out of the 13 total AQPs identified in mammals (AQP1, 2, 3, 4, 5, 6, 8, 9, and 11) have been identified in the human female reproductive system. Hence, these AQPs have significant functions in the female reproductive system, participating in processes such as cervical maturation, vaginal lubrication, implantation, egg transport, inhibition of uterine water, oocyte cryopreservation, follicle maturation, and blastocyst formation.^84^ Specifically, AQP1 may potentially be involved in endometriosis through modulation of the Wnt/β-catenin signaling pathway.^85^ AQP2 is found in the endometrial tissues of patients with endometriosis and EC 2 estrogen-dependent diseases. Another study indicated that the expression of AQP5 might also have a significant role in the pathogenesis of endometriosis. In this context, the downregulation of AQP9 was associated with enhanced cell migration and invasion, mediated by the activation of ERK/p38 MAPK signaling pathways.^86^ AQP2, AQP5, and AQP8 exhibit higher expression levels in eutopic endometrial cells compared to ectopic endometrial cells. This implies that eutopic endometrial cells demonstrate more robust migratory activity than their ectopic counterparts in women with endometriosis.^87^ Therefore, further studies are necessary to elucidate the precise signal-regulatory function of AQPs.^88^

### Aquaporins in Acute Kidney Injury

Acute kidney injury (AKI) is a global syndrome marked by a swift reduction in glomerular filtration, resulting in elevated morbidity and mortality rates.^89^ A growing number of studies indicate that nephritis is one of the principal pathological changes revealed in AKI4. Acute kidney injury is the leading cause of death from sepsis, caused by an abnormal inflammatory response to infection.^90^ In the kidney, 8 AQPs, including AQP1-AQP7 and AQP11, which play a role in tissue development, metabolism, and regulation of urine concentration, are found in different cells and segments.^91^ One study proposed that in a mouse model of sepsis induced by CLP, the expression of AQP2 was downregulated through NF-κB, potentially leading to acute renal failure in sepsis.^59^ Renal ischemia/reperfusion (I/R) injury is a prevalent cause of AKI. Numerous studies have demonstrated a close association between AQP expression and I/R-induced AKI. The urinary exosomal secretion of AQP1 and AQP2 was found to be reduced during the AKI stage and in rats undergoing unilateral and bilateral I/R.^92^ Lei and colleagues^93^ showed that AQP3 knockdown worsened kidney injury by inhibiting MAPK signaling in I/R mice and increasing apoptosis. In a comprehensive analysis of differential gene expression in animals adapted to desert environments, it was revealed that AQP4 expression significantly decreased during acute dehydration. This suggests that AQP4 might contribute to water utilization, potentially playing a role in preventing kidney damage.^94^ Therefore, correction or restoration of AQPs may preserve patients from AKI up to a certain point.

### Aquaporins in Gastritis

Various mechanisms play a role in the pathogenesis of gastritis, depending on the causes. However, pathological consequences such as cell changes (degeneration, apoptosis, and necrosis), inflammation, bleeding, and barrier damage in the gastric mucosa are similar.^95^ Aquaporins in gastric physiology are believed to serve various functions, encompassing gastric juice secretion, water transfer, barrier function, the secretion and absorption of water, and even small solutes through the epithelium.^96^ So far, a minimum of 10 types of AQPs (AQP1-AQP11) have been demonstrated to be found in the human stomach.^97^ Certain AQPs have been identified in cases of chronic gastritis. Specifically, AQP3 mRNA has been observed in patients with chronic atrophic and chronic superficial gastritis. Notably, there is a significantly higher expression of AQP3 in the gastric mucosa of individuals with chronic superficial gastritis when compared to those with chronic atrophic gastritis.^98^ Furthermore, an additional study demonstrated the significance of both AQP1 and AQP4 in preserving mucosal integrity. The study indicated that the expression of these AQPs in the stomach increases during ethanol-induced edema and after gastric injury.^99^ In a separate study, it was demonstrated that AQP3, along with other AQP5, AQP7, and AQP11, exhibited upregulation at the mRNA level in cases of atrophic gastritis.^100^ Thus, these findings suggest that the expression of various AQP subtypes (including AQP1, AQP3, AQP4, AQP5, AQP7, and AQP11) is heightened in the presence of gastritis. Further investigations are warranted to explore their potential utility as diagnostic biomarkers and therapeutic targets in the management of gastritis.

## Conclusion

Inflammation is an intricate mechanism that plays a pivotal role in sustaining mammalian physiology. The involvement of AQPs in numerous vital cellular processes essential for resolving inflammation, including antigen uptake, migration, inflammasome activation, and phagocytosis, along with their participation in various models of inflammatory diseases, positions these membrane proteins as promising targets for drug discovery. Modulating the presence or functionality of AQPs through bioactive compounds or drugs holds the potential to offer novel therapeutic avenues for a range of diseases characterized by disruptions in cellular homeostasis and inflammatory processes. Therefore, research endeavors should concentrate on the creation of AQP blockers and modulators to address therapeutic requirements and enhance our comprehension of the role of AQPs in combating inflammatory diseases.

## Figures and Tables

**Table 1.** Types and Functions of Aquaporins

Types	Function	Localization	Tissue distrubution
**AQP0 **	Aquaporin	plasma membrane	Human lens^19^
**AQP1 **	Aquaporin	plasma membrane	Red blood cells, lungs, brain, kidney
AQP2	Aquaporin	plasma membrane	Kidney^20^
**AQP4**	Aquaporin	plasma membrane	Spinal cord, brain, optic nerve^22^
**AQP5**	Aquaporin	plasma membrane	Respiratory tract^23^
**AQP6 **	Aquaporin	plasma membrane	Renal tract^23^
**AQP8 **	Aquaporin	plasma membrane	lung, liver, stomach, kidneys, pancreas, salivary glands, testicles, epididymis, duodenum, jejunum, placenta and trachea^25^
**AQP3 **	Aquaglyceroporin	plasma membrane	Skin, eyes^21^
**AQP7 **	Aquaglyceroporin	plasma membrane	Skeleteal muscle, heart, testis, kidney, adipose tissue^24^
**AQP9 **	Aquaglyceroporin	plasma membrane	brain, liver, ovary, leukocytes, and testis^23^
**AQP10**	Aquaglyceroporin	plasma membrane	jejunum and duodenum^26^
**AQP11**	Superaquaporin	Intracellular	heart, brain, kidneys, liver, intestine, testes, and adipose tissue^27^
**AQP12**	Superaquaporin	Intracellular	pancreatic acinar cells^28^
